# Genomic and transcriptomic analysis of carbohydrate utilization by *Paenibacillus* sp. JDR-2: systems for bioprocessing plant polysaccharides

**DOI:** 10.1186/s12864-016-2436-5

**Published:** 2016-02-24

**Authors:** Neha Sawhney, Casey Crooks, Virginia Chow, James F. Preston, Franz J. St John

**Affiliations:** Department of Microbiology and Cell Science, University of Florida, Gainesville, FL 32611 USA; Institute for Microbial and Biochemical Technology, Forest Products Laboratory, USDA Forest Service, Madison, WI 53726 USA

**Keywords:** *Paenibacillus* sp. JDR-2, Xylans, Soluble β-glucan, Starch, Bioprocessing systems, Transcriptome, RNA sequencing, Biofuels and chemicals

## Abstract

**Background:**

Polysaccharides comprising plant biomass are potential resources for conversion to fuels and chemicals. These polysaccharides include xylans derived from the hemicellulose of hardwoods and grasses, soluble β-glucans from cereals and starch as the primary form of energy storage in plants. *Paenibacillus* sp. JDR-2 (Pjdr2) has evolved a system for bioprocessing xylans. The central component of this xylan utilization system is a multimodular glycoside hydrolase family 10 (GH10) endoxylanase with carbohydrate binding modules (CBM) for binding xylans and surface layer homology (SLH) domains for cell surface anchoring. These attributes allow efficient utilization of xylans by generating oligosaccharides proximal to the cell surface for rapid assimilation. Coordinate expression of genes in response to growth on xylans has identified regulons contributing to depolymerization, importation of oligosaccharides and intracellular processing to generate xylose as well as arabinose and methylglucuronate. The genome of Pjdr2 encodes several other putative surface anchored multimodular enzymes including those for utilization of β-1,3/1,4 mixed linkage soluble glucan and starch.

**Results:**

To further define polysaccharide utilization systems in Pjdr2, its transcriptome has been determined by RNA sequencing following growth on barley-derived soluble β-glucan, starch, cellobiose, maltose, glucose, xylose and arabinose. The putative function of genes encoding transcriptional regulators, ABC transporters, and glycoside hydrolases belonging to the corresponding substrate responsive regulon were deduced by their coordinate expression and locations in the genome. These results are compared to observations from the previously defined xylan utilization systems in Pjdr2. The findings from this study show that Pjdr2 efficiently utilizes these glucans in a manner similar to xylans. From transcriptomic and genomic analyses we infer a common strategy evolved by Pjdr2 for efficient bioprocessing of polysaccharides.

**Conclusions:**

The barley β-glucan and starch utilization systems in Pjdr2 include extracellular glycoside hydrolases bearing CBM and SLH domains for depolymerization of these polysaccharides. Overlapping regulation observed during growth on these polysaccharides suggests they are preferentially utilized in the order of starch before xylan before barley β-glucan. These systems defined in Pjdr2 may serve as a paradigm for developing biocatalysts for efficient bioprocessing of plant biomass to targeted biofuels and chemicals.

**Electronic supplementary material:**

The online version of this article (doi:10.1186/s12864-016-2436-5) contains supplementary material, which is available to authorized users.

## Background

The bacterium *Paenibacillus* sp. JDR-2 (Pjdr2) originally isolated from sweetgum wood (*Liquidambar styraciflua*) disks exposed to surface soils has been shown to completely utilize the lignocellulosic polymer glucuronoxylan (GX_n_). Previous studies showed that growth on minimal media supplemented with polymeric xylan was preferred to that on simple sugars such as xylose, glucose, or arabinose [1, 2]. These studies indicated that efficient xylan utilization is attributable, in part, to a 157 kDa GH10 β-1,4-endoxylanase (Xyn10A_1_) containing carbohydrate binding modules (CBM) for binding to polysaccharides and surface layer homology (SLH) domains for cell-association. The efficiency of utilization was such that the products of xylan hydrolysis were rapidly assimilated as they were formed [2, 3]. These early findings suggest that Pjdr2 utilizes glucuronoxylan in a vectorial manner with an unidentified mechanism for coupling surface localized polymer hydrolysis to rapid oligoxyloside transport into the cell.

**Fig. 1:**
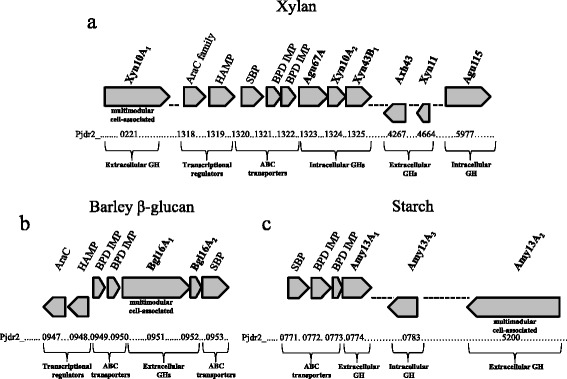
Polysaccharide utilization regulons in Pjdr2. Genomic organization of xylan utilization genes (**a**) assigned a role in xylan utilization in Pjdr2. For a complete list see Additional file 1 [1]. Genetic organization of barley β-glucan (**b**) and starch (**c**) utilization regulons consisting of genes encoding extracellular multi-modular cell-associated or secreted glycoside hydrolases for depolymerization, ABC transporters for assimilation of the generated oligosaccharides, and intracellular glycoside hydrolases for further processing and metabolism. SBP, solute binding protein; IMP, inner membrane protein; BPD, binding protein dependent; GH, glycoside hydrolase. Locus tag annotated as Pjdr2_#### abbreviated to only consist of the numeric portion, ####

PCR screening for genes encoding enzymes typically involved in utilization of GX_n_ led to the identification of the aldouronate utilization gene cluster (Fig. 1a). This cluster of genes encodes three intracellular glycoside hydrolases, a GH67 α-glucuronidase, a GH10 endoxylanase and a GH43 β-xylosidase (Agu67A, Xyn10A_2_ and Xyn43B_1_, respectively), as well as regulatory proteins and ABC (ATP-binding cassette) transporters (Fig. 1a and Additional file 1). Through *in silico* analysis this gene cluster is predicted to contain multiple promoters and catabolite repression elements (cre) although the entire region has only a single detected terminator following the last gene, *xyn43B*_*1*_. Importantly, the aldouronate utilization gene cluster was coordinately regulated with the distally located *xyn10A*_*1*_, supporting the role of this large surface anchored xylanase in membrane localized glucuronoxylan hydrolysis [4]. More recent studies showed that Pjdr2 utilized polymeric GX_n_ at a rate 2.8 times higher than the GX_n_ derived aldouronate, aldotetrauronic acid, suggesting a functional coupling of primary xylan hydrolysis to oligosaccharide transport into the cell [3].

Analysis of the sequenced genome [5] for carbohydrate active enzymes (CAZy) through the CAZy database (http://www.cazy.org/) [6] identified a number of genes encoding enzymes predicted to be involved in xylan utilization. These include five genes encoding GH10 and one encoding GH11 xylanases with expected, and in three cases demonstrated, endo-β-1,4-xylanase activity for xylan main chain hydrolysis. Genes encoding accessory enzymes for xylan depolymerization including β-xylosidases, α-glucuronidases, α-L-arabinofuranosidases, and acetyl esterases are also predicted to be numerous. For example, the genome of Pjdr2 contains 25 genes encoding a broad class of GH43 enzymes including β-xylosidases and α-L-arabinofuranosidases. There are also four genes encoding α-glucuronidases of which three are in the GH115 family and one in the GH67 family. Recombinant enzymes encoded by several of these genes have been characterized [2, 3, 7]. Several other CAZy families commonly involved in xylan utilization are also highly represented. The cumulative potential xylan degrading capacity of Pjdr2 supports its demonstrated abilities for complete xylan utilization.

More recent efforts have focused on transcriptomic analysis of Pjdr2 while growing on xylan substrates. Proteins involved in xylan utilization were identified based on gene expression levels [1]. Primary findings of that study revealed that Pjdr2 secretes only two endoxylanases for xylan depolymerization. The resulting mixture of oligosaccharides generated by Xyn10A_1_ [2] and Xyn11 (unpublished) consists of neutral oligoxylosides and, depending on the xylan type, a mixture of substituted xylooligosaccharides including aldouronates and similarly substituted oligoarabinoxylosides. Genes predicted to encode ABC transporters were found to have increased transcript levels during growth on xylans and it was proposed that these enable transport of this complex mixture of oligoxylosides into the cell for intracellular hydrolysis to monomeric sugars for catabolism [2–4, 8]. The observed high expression level of genes encoding intracellular oligosaccharide degrading accessory enzymes during growth on xylans supported this hypothesis (Additional file 1). Comparative transcriptomics following growth of Pjdr2 on dicot-derived hardwood (sweetgum) GX_n_ and a monocot-derived grass (sorghum) glucuronoarabinoxylan (GAX_n_) indicated that systems with distinct enzymes and ABC transporters are employed for utilization of oligoarabinoxylosides as compared to aldouronates [1].

Based on the earlier physiological characterization and the most recent transcriptomic study, the proposed model for xylan utilization indicates that Pjdr2 relies on transport and intracellular degradation of xylooligosaccharides. This system in Pjdr2 shows similarity to the polymeric sugar utilization by other bacteria including *Geobacillus*, *Thermotoga* and *Clostridium* [9–11] and stands in contrast to the more classical paradigm for fungi that requires complete extracellular conversion of polysaccharides to monosaccharides [12]. The role of the cell-associated endoxylanase in the xylan utilization systems represents an alternative paradigm to that observed in cellulolytic bacteria such as *Clostridium thermocellum* in which glycoside hydrolases comprise a cell-associated complex as opposed to individual enzymes [13–16].

Recent studies show that Pjdr2 is also capable of efficient utilization of other biomass derived polysaccharides including barley β-glucan and starch [17, 18]. Genome analysis indicates that these polysaccharide utilization systems include extracellular glycoside hydrolases with modular architecture for cell-association and carbohydrate binding. We present here an overview of a broad transcriptomic study characterizing Pjdr2 gene regulation in response to growth on barley β-glucan and starch as well as their constituent disaccharide sugars, cellobiose and maltose. The results are additionally considered in regards to the previously studied xylan-utilization system, providing a comparison of these three polysaccharide-utilization systems with respect to transport and catabolism of the products of depolymerization as well as their monosaccharide constituents. Comparison of these three polysaccharide utilization systems of Pjdr2 indicate a reliance upon cell-associated glycoside hydrolases with CBM’s for interacting with polysaccharides and SLH domains for cell-association. Furthermore, identification of 29 genes within the Pjdr2 genome encoding proteins involved in carbohydrate utilization that contain sets of SLH domains supports an evolutionary path leading to the secretion of cell-associated glycoside hydrolases. This system is efficient in the depolymerization of polysaccharides at the cell surface and is found in Pjdr2 as well as related bacteria including *Clostridium* [19–21], *Caldicellulosiruptor* [22] and *Thermoanaerobacter* [23].

## Results and discussion

### Experimental design

For this transcriptome study, we sought a greater understanding of how Pjdr2 utilizes polymeric sugars. Genome analysis and polysaccharide growth studies supported efficient utilization of the polysaccharides soluble β-glucan and starch. Through bioconversion these abundant biomass-derived sugar polymers may contribute to the production of value-added chemical or fuels. To obtain a broad understanding of how these polysaccharides are utilized by Pjdr2, total RNA was prepared from early-mid exponentially growing cultures growing on these polysaccharides as well as their limit enzymatic hydrolysis products and their constituent simple sugars. The sample preparation and RNA-seq data acquisition portions of this work overlap with a recently published xylan utilization transcriptome and the results presented here are compared with this earlier work to provide perspective, draw conclusions and identify themes which define the efficient manner in which Pjdr2 utilizes polysaccharides [1]. The saccharides used in the study that provided the final comparative data set include barley β-glucan (B) and cellobiose (C) representing β-configured glucans, starch (S) and maltose (M) representing α-configured glucans, sweetgum glucuronoxylan (SG) and sorghum glucuronoarabinoxylan (SO) representing different xylan types and the constituent monosaccharides of these polysaccharides, including glucose (G), xylose (X) and arabinose (A). Further, each condition was routinely compared to a yeast extract (YE) control condition which consisted of 0.5 % YE without added carbohydrate and a sweetgum xylan with no YE (SGnoYE) control. Throughout the manuscript where specific genes are considered, their respective transcript levels or their encoded proteins are routinely identified with an accompanying abbreviated locus tag accession number consisting of the four digit number, e. g. the locus tag Pjdr2_0001 would be described as 0001. The total data set normalized RPKM (*R*eads *P*er *K*ilobase per *M*illion reads sequenced) values were compared by fold changes taken from the ratio of the condition in consideration over the YE control condition unless otherwise stated. Data was judged to be significant given a 4-fold change and a *p*-value, < 0.05.

### Genes involved in barley β-glucan utilization

Recent studies have shown that Pjdr2 may utilize soluble β-glucans [18]. Barley β-glucan consists of a linear polysaccharide chain of β-1,4 linked glucose frequently and regularly interrupted with β-1,3-linked glucose [24]. This polysaccharide lacks side chain substitutions such as those found in xylan, hence the extracellular degradation of barley-β-glucan is correspondingly less complex, presumably requiring fewer enzymes. The genome of Pjdr2 encodes three GH16 enzymes (genes 0951, 0952, and 0824) annotated as licheninases or laminarinases through domain analysis. All three enzymes are predicted to be secreted and while one consists of a singular GH16 catalytic module, the other two have an extensive multimodular architecture. Both of these modular enzymes (genes 0951 and 0824) contain triplicate N-terminal SLH domains, presumably for cell surface localization, and multiple CBM’s similar to those observed for the Xyn10A_1_ enzyme involved in xylan utilization (Fig. 4).

During growth on barley-β-glucan, the genes encoding the multimodular Bgl16A_1_ (gene 0951) and the non-modular Bgl16A_2_ (gene 0952) increased 80-fold and 25-fold respectively compared to growth on the yeast extract control (YE) not supplemented with carbohydrate (Table 1). The *bgl16A*_*3*_ gene (0824) encoding the second large multimodular GH16 enzyme is expressed only at low levels on all substrates tested. Bgl16A_1_ shares 34 % amino acid similarity with the catalytic domain of the GH16 laminarinase from *Thermotoga maritima* (UniProt accession: Q9WXN1) [25] and Bgl16A_2_ shares 71 % similarity to a probable licheninase from *Bacillus subtilis* (UniProt accession: P04957) (Table 2) [26]. In support of these annotations, recombinant Bgl16A_1_ has been shown to have activity against barley β-glucan and laminarin as both substrates contain the requisite β-1,3-glucan linkage while recombinant Bgl16A_2_ shows its highest activity on barley β-glucan [18].

**Table 1:** Expression analysis of polysaccharide processing genes during growth on barley β-glucan, starch and maltose

Family^a^	LT^b^	Protein product	Name^c^	SP^d^	Fold change^e^					Linear RPKM Values^f^					
					B/YE	C/YE	G/YE	S/YE	M/YE	B	C	G	S	M	YE
Barley β-glucan utilization															
GH16	0951	laminarinase	**Bgl16A**_**1**_	Yes	**80.3**	0.2	0.2	0.4	0.1	170.2	0.4	0.5	0.8	0.3	2.1
GH16	0952	endo-β-1,3-1,4 glucanase	**Bgl16A**_**2**_	Yes	**24.5**	0.2	0.2	0.3	0.1	124.1	1.1	0.8	1.5	0.6	5.1
GH16	0824	laminarinase	**Bgl16A**_**3**_	Yes	0.4	0.5	0.6	0.6	0.6	1.7	2.2	2.7	2.4	2.4	4.1
GH3^g^	0317	glycoside hydrolase		No	2.7	2.7	0.5	NS	2.4	379.0	376.6	70.7	156.1	338.1	141.5
Barley β-glucan induced xylanases															
GH67	1323	α-glucuronidase	**Agu67A**	No	**20.6**	0.8	0.4	0.6	0.4	120.2	4.4	2.1	3.5	2.5	5.8
GH8	1182	exooligoxylanase	Xyn8	No	**39.3**	0.4	0.4	0.7	0.5	297.2	3.3	3.1	5.1	3.5	7.6
GH11	4664	endoxylanase	Xyn11	Yes	3.7	0.4*	0.2	0.3*	0.2	7.5	0.8	0.4	0.5	0.4	2.1
GH10	0221	endoxylanase	**Xyn10A**_**1**_	Yes	**8.8**	0.2	0.1	0.3	0.1	52.0	1.2	0.8	1.6	0.6	5.9
GH10	1324	endoxylanase	**Xyn10A**_**2**_	No	**24.0**	0.8*	0.5	0.6*	0.6	185.3	6.2	3.5	4.7	4.4	7.7
GH43	1325	xylosidase	Xyn43B_1_	No	**19.6**	0.9*	0.5	0.7*	0.8*	222.1	9.9	5.9	7.6	8.6	11.3
GH43	0750	xylosidase	Xyn43B_2_	No	3.2	**176.6**	NS	NS	NS	5.0	276.7	2.7	2.6	1.8	1.6
GH43	1907	xylosidase	Xyn43B_3_	No	**111.5**	2.3	NS	NS	0.6	1410.9	29.6	12.2	13.5	7.6	12.7
Starch utilization															
GH13	0774	α-amylase	Amy13A_1_	Yes	0.2	0.2	0.1	**114.1**	**69.7**	2.0	1.8	1.3	1127.3	688.4	9.9
GH13	5200	α-amylase	Amy13A_2_	Yes	0.2	0.2	0.2	**56.8**	**4.2**	1.5	1.5	1.2	395.7	29.3	7.0
GH13	0783	α-amylase	Amy13A_3_	No	1.5	NS	NS	**112.2**	**95.6**	7.8	5.7	6.5	584.3	497.9	5.2
GH13	1045	α-amylase	Amy13A_4_	No	NS	2.7	3.8	NS	NS	2.6	4.7	6.6	1.5	1.4	1.7
GT^g^	1149	α-glucan phosphorylase	MalP	No	0.3	0.1	0.5	NS	0.2	2.4	0.9	0.7	6.6	1.5	8.9
Maltose utilization															
ND	5587	oxidoreductase	ThuB	No	NS	NS	NS	NS	**8.8**	7.4	7.0	7.3	9.0	73.8	8.4
GATase1	5588	hypothetical protein	ThuA	No	0.7	0.7	NS	NS	**8.1**	7.1	2.6	2.5	2.5	30.3	3.8

^a^GH, glycoside hydrolase; GT, glycosyltransferase; ND, not determined; GATase1, type 1 glutamine amidotransferase (GATase1)-like domain

^b^ LT, locus tag annotated as Pjdr2_#### abbreviated to only consist of the numeric portion, ####

^c^The name assigned to gene candidates with enzymes characterized in our laboratory in bold

^d^SP, sequence encodes a predicted signal peptide for secretion

^e^Transcript levels of candidate genes that were expressed 2-fold greater (underlined) and those that were expressed 4-fold greater (bold) than the yeast extract without carbohydrate growth are indicated. The growth substrates are shown as follows: B, barley β-glucan; C, cellobiose; G, glucose; S, starch; M, maltose; YE, yeast extract. Significance of fold change data is judged by having a *p*-value no more than 0.01. Data with *p*-values between 0.01 and 0.05 are denoted with an asterisk, and those with *p*-values greater than 0.05 are designated as not significant (NS)

^f^RPKM values are defined as *R*eads *P*er *K*ilobase per *M*illion reads sequenced

^g^Gene 0317 and gene 1149 are included in this table as genes of interest in barley β-glucan and starch utilization pathways, respectively. Gene 0317 is increased 5.4-fold on barley β-glucan (*p*-value < 0.0002) and gene 1149 is increased 9.4-fold on starch (*p*-value < 0.037) relative to growth on glucose

**Table 2:** Orthologs of translated sequences encoded by candidate genes from Pjdr2

LT^a^	Protein	Orthologue (UniProt accession)	Identity (%)^b^
0951	multimodular Bgl16A_1_	laminarinase from *Thermotoga maritima* (Q9WXN1)	34
0952	Bgl16A_2_	probable lichinase from *Bacillus subtilis* (P04957)	71
0774	Amy13A_1_	extracellular amylase from *Bacillus megaterium* (P20845)	46
5200	multimodular Amy13A_2_	amylopullanase from *Thermoanaerobacter pseudethanolicus* (P38939)	33
0783	Amy13A_3_	intracellular maltogenic amylase from *B. subtilis* (O06988)	47
0771	SBP^c^	maltodextrin binding protein from *Bacillus subtilis* 168 (O06989)	33
1340	SYM^d^	AraE xylose and arabinose symporter in *B. subtilis* (X98354)	49

^a^LT, locus tag annotated as Pjdr2_#### abbreviated to only consist of the numeric portion, ####

^b^amino acid sequence identity

^c^SBP, solute binding protein

^d^SYM, symporter

Hydrolysis of barley β-glucan with GH16 laminarinase and licheninase enzymes is expected to liberate β-1,3/1,4 mixed linkage glucooligosaccharides. Increased transcript levels for two predicted ABC transporter gene cassettes were observed during growth on barley β-glucan compared to the YE control. The first cassette consisting of genes 0949, 0950 and 0953, flanks the enzyme encoding genes *bgl16A*_*1*_ and *bgl16A*_*2*_ described above and showed greater than a 1200-fold increase in transcript levels during growth on barley β-glucan (Table 3). These barley β-glucan utilization genes constitute an apparent operon specifically responsive to growth on soluble β-1,3 (4)-glucans and no other tested substrate. This operon is directly linked to a β-glucan-responsive set of putative transcriptional regulators (genes 0947 and 0948) located immediately upstream but transcribed in the opposite direction. Together, these seven genes, 0947 through 0953, constitute the glucan utilization gene cluster (Fig. 1b). The second ABC transporter gene cassette consisting of genes 5314, 5315, and 5316 has increased expression on barley β-glucan and was also increased on xylan [1]. This overlapping regulation will be discussed below.

**Table 3:** Expression analysis of genes encoding ABC transporters during growth on barley β-glucan, starch, cellobiose and maltose

LT^a^	Protein product^b^	Fold change^c^					Linear RPKM Values^d^					
		B/YE	C/YE	G/YE	S/YE	M/YE	B	C	G	S	M	YE
0472	BPD transport system IMP	NS	**23.1**	**18.3**	NS	2.7*	1.7	27.7	22	1.1	3.3	1.2
0473	BPD transport system IMP	NS	**20.8**	**18.9**	NS	3	1.8	31	28.2	1.9	4.4	1.5
0474	extracellular SBP	0.7	**7.4**	**9.6**	NS	NS	6.5	71.7	92.8	8.6	13.4	9.7
0728	extracellular SBP	0.3	**7.2**	0.2	NS	0.2	7.1	173.8	4	19	6	24
0729	BPD transport system IMP	0.4	**8.6**	0.5	NS	0.4	6.2	119.1	6.5	12.8	5.1	13.9
0730	BPD transport system IMP	0.5	**8.8**	0.5	NS	0.5	7.3	134.5	8.2	16.4	7.7	15.2
0771	extracellular SBP	0.1	0	0	**86.4**	**24.1**	3.9	2.4	2.6	5524	1543.1	64
0772	BPD transport system IMP	0.1	0.1	0.1	**94.3**	**34.7**	1.1	0.8	0.9	1174	432.6	12.5
0773	BPD transport system IMP	0.1	0.1	0.1	**134.8**	**58.3**	1.3	0.8	0.8	1390	600.5	10.3
0949	BPD transport system IMP	**1451.2**	NS	NS	NS	NS	3602	2.1	2	2.2	3.1	2.5
0950	BPD transport system IMP	**1498.5**	1.7	NS	NS	NS	5522	6.4	3.5	4.8	4.9	3.7
0953	extracellular SBP	**1220.8**	2	NS	NS	0.6	5381	8.8	3.5	5	2.6	4.4
1320	extracellular SBP	0.2*	0.6	0.1	0.4*	0.1	4.2	11.5	1.4	6.9	1.8	18.6
1321	BPD transport system IMP	0.1	NS	0.1	0.6*	0.1	0.7	4.8	0.5	3	0.5	5
1322	BPD transport system IMP	**15.1**	NS	0.3	0.6	0.4	111.3	8.1	2.6	4.6	3.2	7.4
3245	periplasmic binding protein	3.5	2.6	2.3	2.3	**10.9**	158.4	119.1	103.9	107	500.4	45.7
3597	extracellular SBP	**15**	0.5	0.6*	2.4*	NS	19.9	0.7	0.8	3.2	1.1	1.3
5314	BPD transport system IMP	**39.9**	0.4	0.2	0.5	0.3	426.6	4.4	2.1	5.8	2.7	10.7
5315	BPD transport system IMP	**42.1**	0.4	0.2	0.4*	0.2	379.7	3.2	1.4	3.7	1.4	9
5316	extracellular SBP	**31.1**	0.2	0.1	0.3	0.1	797.5	5.4	1.9	7.5	1.6	25.7
5589	extracellular SBP	0.5	0.5	0.5*	NS	**9.8**	2.2	2.1	2.3	3.3	42.9	4.4
5590	BPD transport system IMP	NS	NS	NS	NS	**10.9**	1.1	1.2	1.8	1.4	15.3	1.4
5591	BPD transport system IMP	0.5	NS	NS	NS	**14.9**	0.4	0.6	0.9	1.1	13.7	0.9
5596	BPD transport system IMP	3.1	**44.8**	0.6	0.6	0.4	19.7	281.7	3.5	3.5	2.4	6.3
5597	BPD transport system IMP	3.3	**52.2**	0.5*	NS	0.2	14.1	219.5	2.1	2.2	1	4.2
5598	extracellular SBP	**4.4**	**54.5**	0.1	0.2*	0.1	40.3	494.9	1.1	2.3	0.9	9.1
5960	BPD transport system IMP	**15.3**	**154.6**	**15.2**	NS	0.5	269.7	2721	267.6	22.6	8.8	17.6
5961	BPD transport system IMP	**16**	**145.5**	**12.7**	NS	0.5	227.1	2062	179.8	22.6	7.8	14.2
5962	extracellular SBP	**18.1**	**118.5**	**12.2**	NS	0.3	620.6	4070	419.6	33.1	9.4	34.3

^a^LT, locus tag annotated as Pjdr2_#### abbreviated to consist only of the numeric portion, ####

^b^SBP, solute binding protein; IMP, inner membrane protein; BPD, binding protein dependent

^c^Transcript levels of candidate genes that were expressed 2-fold greater (underlined) and those that were expressed 4-fold greater (bold) than the yeast extract without carbohydrate are indicated. The growth substrates are shown as follows: B, barley β-glucan; C, cellobiose; G, glucose; S, starch; M, maltose; YE, yeast extract. Significance of fold change data is judged by having a *p*-value no more than 0.01. Data with *p*-values between 0.01 and 0.05 are denoted with an asterisk, and those with *p*-values greater than 0.05 are designated as not significant (NS)

^d^RPKM values are defined as *R*eads *P*er *K*ilobase per *M*illion reads sequenced

Comparison of the barley β-glucan utilization system described here to the xylan utilization system described previously [1] implies a missing enzymatic component for barley β-glucan utilization. Pjdr2 appears to transport the mixed linkage oligosaccharide products of the two secreted GH16 endoglucanases in a manner similar to xylan utilization. However, unlike the defined intracellular oligosaccharide processing in the xylan utilization systems, there is no clear evidence for increased expression of genes encoding enzymes for intracellular hydrolysis of glucooligosaccharides contributing to the barley β-glucan utilization system. Genome analysis has identified several genes encoding enzymes which could be involved in the further processing of intracellular glucooligosaccharides (e.g. the genome encodes fifteen GH3 enzymes), but none of these genes are confidently assigned to this role based on the transcriptomic data. One candidate, gene 0317, encoding an intracellular GH3 β-glucosidase attains elevated transcript levels with growth on barley-β-glucan and cellobiose. The comparatively high expression level on YE resulted in a limited relative increase of just 2.7-fold with barley β-glucan, although compared to growth on arabinose or glucose this gene had a 10 and 5.4-fold increase in expression, respectively (Table 1).

### Genes with increased expression during growth on cellobiose

Cellobiose is thought to represent a primary limit product of mixed linkage β-glucan utilization and was chosen for study to discriminate between utilization of the barley β-glucan polymer and its hydrolysis products. Cellobiose as a growth substrate results in increased expression of several genes encoding putative ABC transporters in Pjdr2. The genes 5960, 5961 and 5962 show greater than 118-fold increase in expression on cellobiose relative to YE. Both glucose and barley β-glucan also induce the genes encoding this transporter although to a lower extent (Table 3). From the transcriptome data it is not known precisely how cellobiose is converted to glucose for entry into glycolysis. As detailed above for the intracellular processing of glucooligosaccharides resulting from barley β-glucan utilization the putative intracellular GH3 β-glucosidase (gene 0317) may serve this role. This gene is expressed on cellobiose at nearly the same increased level as found with growth on barley β-glucan relative to other sugars, but not YE (Table 1). In addition, gene 0750 encoding a putative intracellular β-xylosidase (Xyn43B_2_) that was earlier predicted to be involved in xylan utilization due to its 100-fold increase on xylan relative to YE (Additional file 1) is found in this work to be increased 177-fold during growth on cellobiose [1] (Table 1). This gene may encode the enzyme primarily responsible for hydrolysis of cellobiose. If so, this putative xylosidase either has dual substrate specificity or it actually encodes a GH43 β-glucosidase the expression of which is induced by cellobiose and to a lesser extent xylobiose. The GH43 family does not as yet contain an enzyme with a reported β-glucosidase activity. The expression of *xyn43B*_*2*_ is also increased on barley β-glucan by 3-fold (Table 1) relative to YE and may contribute as well to the hydrolysis of the glucooligosaccharides derived from this polymer.

### Genes involved in starch utilization

Pjdr2 grows very efficiently on starch [17]. Utilization of this α-1,4-linked glucose storage polysaccharide appears similar to barley β-glucan as this polysaccharide is also chemically simple relative to xylans with fewer enzymes required for degradation to glucose. The genome of Pjdr2 encodes four GH13 amylases. Three of these, Amy13A_1_, Amy13A_2_, and Amy13A_3_ have significantly increased transcript levels ranging from 55-fold to over 100-fold increased expression during growth on starch (Table 1). Both Amy13A_1_ (gene 0774) and Amy13A_2_ (gene 5200) are predicted to be secreted and primarily responsible for endo-hydrolysis of native starch. The *amy13A*_*2*_ gene encodes a large multimodular enzyme including SLH domains and CBM’s for cell surface proximal substrate localization (Fig. 4) while *amy13A*_*1*_ encodes only a catalytic domain. The starch utilization system in Pjdr2 also has a predicted intracellular amylase, Amy13A_3_ (gene 0783) presumably to complete the degradation of the transported, intracellular maltodextrins.

Amy13A_1_ shares 46 % amino acid sequence identity with the extracellular amylase from *Bacillus megaterium* (UniProt accession: P20845) (Table 2) [27, 28] and Amy13A_2_ shares 33 % identity over a large portion of its modular sequence with an amylopullanase from *Thermoanaerobacter pseudethanolicus* (UniProt accession: P38939) [29]. Amy13A_3_ shares 47 % amino acid identity with an intracellular maltogenic amylase from *B. subtilis* (UniProt accession: O06988) [30] where it is thought to function in the conversion of maltotriose and larger maltodextrins to maltose and glucose (Table 2).

A single ABC transporter gene cassette showed increased transcript levels during growth on starch relative to YE (Table 3). The genes for this transporter (genes 0771, 0772 and 0773) are just upstream of *amy13A*_*1*_ (0774) and form a predicted operon (Fig. 1c). The solute binding protein (gene 0771) of this transporter shares 33 % amino acid identity with a maltodextrin binding protein from *Bacillus subtilis* 168 (Table 2) [31]. This putative maltose/maltodextrin ABC transporter gene cassette was shown to be markedly up-regulated during growth on xylans (Fig. 3b, Additional file 2) [1]. However, the genomic localization of this ABC gene cluster within a predicted operon containing the gene encoding extracellular amylase suggests its primary function is that of a maltodextrin transporter (Fig. 1c). This overlap in regulation will be further discussed below.

The high amino acid sequence identity between Amy13A_3_ and the maltogenic amylase from *B. subtilis* suggest that this enzyme might process transported maltodextrins to glucose and maltose [30]. As a component of a complete starch utilization system, gene 1149 encodes a putative α-glucan phosphorylase (MalP) allowing for phosphorolytic cleavage of intracellular maltose [32, 33]. Expression levels of this gene on starch compared to YE yielded insignificant results (*p*-value, 0.403), but a 9.1-fold transcript increase is observed relative to growth on glucose (*p*-value, 0.004) (Table 1). Transcript data for the fourth predicted amylase encoding gene, 1045, was not considered statistically significant (*p*-value, 0.635) and did not appear to exhibit dynamic regulation on starch, barley β-glucan or xylan and linear RPKM values were comparatively low (1.2-1.6).

### Genes with increased expression during growth on maltose

As the primary hydrolysis limit product of starch, maltose was included in this study to distinguish physiological features for efficient starch utilization. The transporter genes described above as part of the putative starch utilization operon are also upregulated. In addition, genes 5589, 5590 and 5591 encoding a second ABC transporter are up-regulated approximately 10-fold on maltose over YE (Table 3). Once internalized, maltose would be expected to follow the pathway similar to that predicted in starch utilization; however, for this growth condition expression of the gene encoding the MalP protein is not increased relative to any other growth condition.

This finding reveals a difference between the intracellular processing of maltodextrins derived from starch hydrolysis by the surface localized multimodular Amy13A_2_ and maltose directly assimilated. A focused search failed to identify homologs of genes known for the conversion of maltose or maltose-6-phosphase (e. g. glucose phosphorylase or 6-phospho-alpha-glucosidase). Two other genes upstream of those encoding the maltose specific transporter identified above code for proteins annotated as an oxidoreductase (gene 5587) and a hypothetical protein (gene 5588) and the predicted operon appears related to the *thuAB* encoding operon involved in trehalose utilization in *Agrobacterium tumefaciens* [34] (Table 1). This suggests that Pjdr2 converts maltose to 3-keto-maltose.

### Monosaccharide assimilation and metabolism

From genome analysis [5], intracellular metabolism of the hexose, glucose, and the pentoses, xylose and arabinose, are expected to follow through the Embden-Meyerhof-Parnas (EMP) pathway and pentose phosphate pathway (PPP), respectively, for entrance into the tricarboxylic acid (TCA) cycle. Following transport of arabinose through the previously identified arabinose responsive ABC transporter [1], this sugar may be converted to ribulose-5-phosphate by the arabinose isomerase and ribulose kinase enzymes. In Pjdr2, the gene 2502 (Table 4) attains a 24-fold increase in transcript level with growth on arabinose and 4.9-fold on sorghum MeGAX_n_ (Additional file 2). Based on transcript levels the candidate ribulose kinase enzyme is encoded by gene 4209. This enzyme is a distant homolog (~21 % ID) to the AraB protein which is a component of the L-arabinan utilization system of *Geobacillus stearothermophilus* [35] (Table 2). Transcript levels of gene 4209 are increased 17-fold on arabinose (Table 4) and 3-fold on sorghum MeGAX_n_ relative to YE (Additional file 2). This gene does not show an increased transcript level on other carbohydrate growth conditions used in this study. The genes 0977, 0978 and 0979 encoding an ABC transporter are primarily responsive to xylose resulting in an average of 135-fold increase in transcript level relative to YE (Table 4). These genes also showed significant but much lower fold increases on glucose and arabinose. Additionally, a predicted symporter encoded by gene 1340 shares 49 % identity with the AraE xylose and arabinose symporter in *B. subtilis* (Table 2) [36]. Expression of this gene is responsive to xylose resulting in a 162-fold increase. This gene is also expressed on cellobiose (Additional file 2), glucose and arabinose although to a much lower extent than observed on xylose (Table 4). Conversion of xylose to xylulose-5-phosphate follows a similar path as arabinose since genes encoding xylose isomerase (gene 5159) and xylulose kinase (gene 5158) result in nearly a 100-fold and 63-fold increase in expression, respectively, on xylose compared to YE controls (Table 4). Growth on xylans also resulted in transcript increases of 35-fold and greater for these two genes (Additional file 2).

**Table 4:** Regulation of genes involved in monosaccharide transport and introduction into metabolic pathways

LT^a^	Protein product^b^	Fold change^c^			Linear RPKM Values^d^			
		G/YE	X/YE	A/YE	G	X	A	YE
Monosaccharide metabolism								
0170	glucokinase	NS	NS	NS	119.2	140.7	131.2	128.5
2502	arabinose isomerase	0.5	0.6	**23.8**	81.6	96.3	4117.9	173.0
4209	ribulose kinase	0.6	NS	**17.1**	13.0	20.9	369.6	21.7
5159	xylose isomerase	0.4	**99.9**	0.5	21.3	6046.1	30.9	60.5
5158	xylulose kinase	0.4	**62.9**	**19.0**	16.9	2951.2	19.0	46.9
Monosaccharide transporters								
0472	BPD transport system IMP	**18.3**	**6.4**	**16.8**	22	7.7	20.2	1.2
0473	BPD transport system IMP	**18.9**	**6.0**	**13.9**	28.2	9.0	20.8	1.5
0474	extracellular SBP	**9.6**	3.1	**5.6**	92.8	30.4	54.2	9.7
0661	extracellular SBP	0.4*	NS	**1917.4**	1.4	4.8	7460.5	3.9
0662	NBD	NS	NS	**1267.6**	1.0	2.1	3374.4	2.7
0663	BPD transport system IMP	0.2	0.6	**1301.2**	0.8	2.0	4343.5	3.3
0977	extracellular SBP	**16.8**	**190.6**	**8.3**	154.2	1747.9	76.2	9.2
0978	NBD	**15.2**	**102.1**	**5.9**	80.2	538.0	30.9	5.3
0979	BPD transport system IMP	**17.6**	**111.9**	**6.4**	105.9	671.5	38.7	6.0
1340	symporter	**39.1**	**162.0**	**14.0**	120.5	499.9	43.4	3.1
2400	extracellular SBP	**4.8**	3.7	3.8	244.9	188.0	193.4	51.1
2401	NBD	**4.3**	1.9	2.3	93.8	41.5	49.4	21.9
2402	BPD transport system IMP	**4.8**	3.3	3.5	116.1	79.2	84.0	24.0

^a^LT, locus tag annotated as Pjdr2_#### abbreviated to consist only of the numeric portion, ####

^b^SBP, solute binding protein; IMP, inner membrane protein; BPD, binding protein dependent; NBD, nucleotide binding domain

^c^Transcript levels of candidate genes that were expressed 2-fold greater (underlined) and those that were expressed 4-fold greater (bold) than the yeast extract without carbohydrate are indicated. The growth substrates are shown as follows: G, glucose; X, xylose; A, arabinose; YE, yeast extract. Significance of fold change data is judged by having a *p*-value no more than 0.01. Data with *p*-values between 0.01 and 0.05 are denoted with an asterisk, and those with *p*-values greater than 0.05 are designated as not significant (NS)

^d^RPKM values are defined as *R*eads *P*er *K*ilobase per *M*illion reads sequenced

While the genes that encode the transporters that import xylose and arabinose can be identified based on homology and increased transcript levels, a system for efficient glucose assimilation is less apparent. Genes encoding three putative ABC transporters showed increased transcript levels with growth on glucose, but for only two of these ABC transporters (genes 0472, 0473 and 0474 and genes 2400, 2401 and 2402) is it possible that glucose may be the target sugar for transport. For both of these transporter gene sets transcript is increased not only on glucose, but also similarly increased on arabinose, xylose and cellobiose (Tables 3 and 4). The 0472–0474 gene set is increased more significantly at approximately 20-fold relative to the YE control, while the 2400–2402 gene set is just greater than the significance cutoff of 4-fold (Table 4 and Additional file 2). The third ABC transporter gene set whose transcript is significantly increased with growth on glucose (genes 0977, 0978 and 0979) is assigned as a xylose transporter. While its expression is significant with growth on glucose, it is very low relative to growth on xylose.

Analysis for phosphotransferase systems (PTS) reveals two operons (gene sets 2007–2010 and 6221–6226) encoding all protein components of a complete PTS system. Based on homology, the 6221–6226 gene set appears very likely to be a mannitol transport system, while the 2007–2010 set encodes a EIIA component (gene 2010) which is annotated as a glucose superfamily transporter, and a separate protein product (gene 2009) encoding the EIIBC components annotated as an N-acetylglucosamine specific transporter (Additional file 2). None of the genes encoding the complete PTS system components have increased transcript responsive to growth on glucose relative to YE. Interestingly, two unlinked PTS system components (gene 3804 annotated as an Enzyme I complex and gene 0174 annotated as HPr phosphocarrier protein) have relatively high constitutive expression levels, but their roles are unclear (Additional file 2). The analysis for potential glucose specific transporters is not conclusive from this data. Once transported into the cell, conversion of glucose to glucose-6-phosphate for entry into glycolysis appears to be mediated by only a single enzyme: a glucokinase (gene 0170) which yields an average RPKM value of 128 ± 15 over all the tested growth conditions (Table 4). This physiological data underscores original research which showed that Pjdr2 does not efficiently utilize simple sugars in minimal salt media [2].

### Overlapping regulation: starch > xylan > soluble β-glucan

**Fig. 2:**
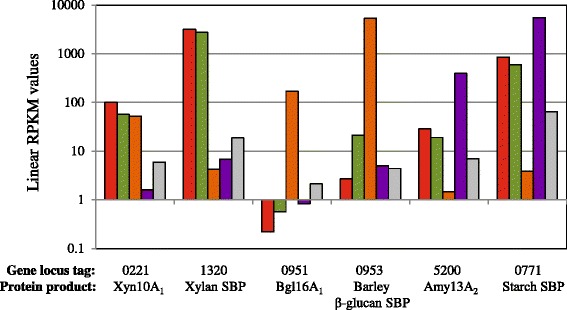
Comparison of gene expression of surface localized substrate specific glycoside hydrolases and their regulon associated solute binding protein. RPKM (*R*eads Per *K*ilobase *p*er *M*illion reads sequenced) values from transcriptomic studies following Pjdr2 growth on sweetgum GXn (SG), sorghum GAXn (SO), barley β-glucan (B) or starch (S) for the genes encoding the large multimodular surface anchored glycoside hydrolase and ABC transporter solute binding protein (SBP) which represents regulation for each of the three polysaccharides. A culture containing only 0.5 % yeast extract without carbohydrate (YE) served as control for comparison. Locus tag annotated as Pjdr2_#### abbreviated to only consist of the numeric portion, ####

**Fig. 3:**
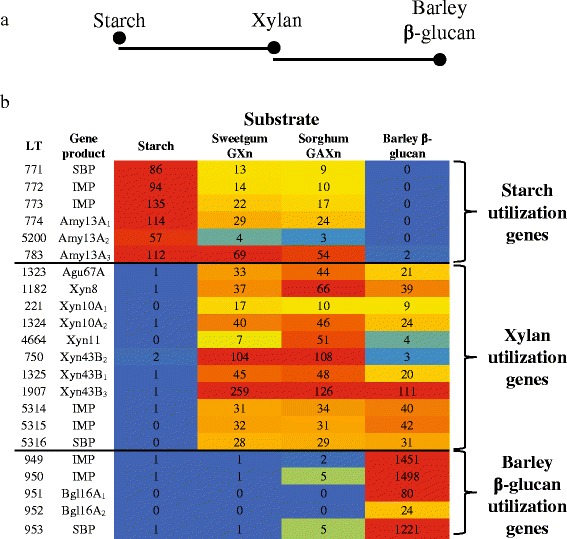
Overlapping regulation of polysaccharide utilization genes in Pjdr2. Schematic representation (**a**) of the regulatory connections between the studied polysaccharide substrates. Growth condition responsive genes (**b**) for barley β-glucan, starch and xylans were compared by hierarchical clustering relative to expression on the yeast extract control. High expression, red; low expression, blue. LT, Locus tag annotated as Pjdr2_#### abbreviated to only consist of the numeric portion, ####

Unexpectedly, the combined data for barley β-glucan, starch and xylan reveals a regulatory connection for utilization of these polymers. This can be seen in quantitative comparisons of the expression of genes encoding the secreted multimodular GH16, GH13, and GH10 endolytic enzymes and those encoding their associated substrate binding proteins that serve as a representative of the specific ABC transporter for the saccharides generated by these enzymes on the cell surface (Fig. 2). Growth on xylans, both GX_n_ and GAX_n_, supports the enhanced expression of genes associated with utilization of xylans and starch but not those associated with the utilization of soluble β-glucan. While barley β-glucan induces genes related to its extracellular degradation and assimilation, these results show that it also induces 8 of the 13 glycoside hydrolase genes involved in xylan utilization (Table 1) [1]. Furthermore, while growth on xylan does not induce any soluble β-glucan utilization genes it does induce genes encoding all of the GH13 α-amylases and the ABC transporter considered to be involved in starch depolymerization and transport for utilization (Fig. 3b and Additional file 2) [1] with the exception of the putative α-glucan phosphorylase gene, *malP*. Following growth on starch, Pjdr2 does not induce genes for either xylan or barley β-glucan utilization. These relationships are represented in the heat map shown in Fig. 3b in which expression of genes encoding ABC transporter proteins as well as accessory enzymes for intracellular metabolism of assimilated oligosaccharides are shown. These findings may be due to a metabolic substrate preference in a manner similar to glucose mediated catabolite repression, or result from evolved enzyme systems for utilization of polysaccharides that are typically associated. In cereal grains, these three carbohydrates can be found together, with xylan and β-glucan localized more to the cell wall and outer layers, and the starch consolidated in the endoplasm [37]. The model that currently describes this relationship (Fig. 3a) can be described as starch first, xylan second and barley β-glucan third. From the observed coordinate gene expression, Pjdr2 appears prepared to utilize multiple polysaccharides (Fig. 3b).

In consideration of the expression of the genes encoding intracellular xylanases, e.g. Xyn43B_1_ and Xyn8 [1] on barley β-glucan, it is possible that these enzymes may have a bifunctional role in the intracellular hydrolysis of β-glucooligosaccharides, thereby, providing an additional route for the intracellular processing of the barley β-glucan derived glucooligosaccharides and cellobiose.

Other genes involved in transport also show overlapping regulation. The predicted ABC transporter previously annotated as a “multiple sugar transport system” consisting of the genes 5314, 5315 and 5316 is shown to have increased expression on barley β-glucan (Table 3) similar to that observed for xylan [1]. This is the only ABC transporter gene set that follows the pattern of expression during growth on barley β-glucan as that observed for the xylan specific glycoside hydrolase genes (Fig. 3b), supporting the possibility that it might be bifunctional in substrate recognition. One other gene encoding an ABC transporter component also follows this pattern. Gene 1322 (Table 3) encoding an inner membrane component, UgpE (BPD transport system IMP, Fig. 1a), of the aldouronate utilization gene cluster [1, 4] has a markedly increased transcript level (15-fold) on barley β-glucan. Studies are underway to elucidate these overlapping regulatory connections.

### Overlapping regulation: cellobiose and xylobiose

Some genes with increased transcript levels during growth on cellobiose were found to also have increased expression levels with growth on xylans. The gene cluster 5596, 5597 and 5598 (Table 3) encodes an ABC transporter annotated as an “unknown carbohydrate transporter” and has been assigned a potential role in xylan utilization based on increased transcript levels (Additional file 1) [1]. Analysis of growth on cellobiose indicates these genes are expressed at a level comparable to that on xylan. A corollary to this finding is the observation that growth on cellobiose also resulted in increased transcript levels for genes 0728, 0729 and 0730 encoding an ABC transporter previously assigned a putative function in xylooligosaccharide (*X*_2_ and X_3_) transport [1]. From the similar level of expression on both xylan and cellobiose, it is proposed that these transporters may be specific for disaccharides such as cellobiose and the primary neutral product of enzymatic xylan hydrolysis, xylobiose. These findings indicate that the transporters of β-configured oligosaccharides may be promiscuous in their substrate recognition.

### Proteins with SLH domains

Enzyme systems utilized by Pjdr2 for the extracellular processing of xylan, barley β-glucan and starch share a common theme. These systems include extracellular cell-associated multimodular glycoside hydrolases to generate oligosaccharides that are released in close proximity to the bacterial cell wall. This functionality is mediated by surface layer homology (SLH) domains that anchor the enzymes to the cell surface [2, 38, 39] and carbohydrate binding modules (CBM) that presumably associate the enzyme with the target polysaccharide [40]. These cell-surface proximal oligosaccharides are then efficiently transported with substrate specific ABC transporters and further hydrolyzed within the cell to monosaccharides for introduction into catabolism. This surface localization represents a strategy for competitive utilization of these polysaccharides. As part of this work we sought to define the roles of SLH domains in Pjdr2 in the processing of plant polysaccharides.

**Fig. 4:**
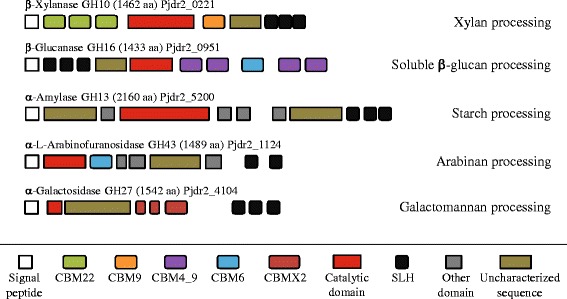
Multimodular cell-associated enzymes from Pjdr2. A diagram representing the domain architecture of the three surface anchored enzymes central to the utilization of xylan, barley β-glucan and starch as reported in this work along with two others, further representing the broad use of SLH mediated surface localization of enzymes for polysaccharide assimilation in Pjdr2 as demonstrated in Table 5. Coding sequence locus tag accession numbers are provided as Pjdr2_####

In total, there are 77 genes encoding proteins with regions homologous with SLH domains. Of these, 73 have two or more consecutive SLH domains which is the minimum set thought to be required for tight binding to the cell wall [41]. Of the 77 SLH domain containing proteins, 29 are predicted to be involved in carbohydrate processing (Table 5) as indicated through domain analysis. In this smaller set, the average calculated protein size is nearly 193 kDa and the average predicted pI is 4.74 with a standard deviation of just 0.10. Domain and BLASTp analysis (Table 5) shows the diversity of functions of associated carbohydrate active enzymes among these SLH proteins (Table 5). In the current transcriptomic data set, only a single SLH-bearing gene has been identified being involved in the catalysis of either xylan (gene 0221), barley β-glucan (gene 0951) or starch (gene 5200) utilization from the 29 identified SLH-encoding carbohydrate processing genes (Table 5). Of the other SLH domain containing proteins in this list it can be seen that Pjdr2 may utilize numerous other polysaccharides with the same strategy. Some of these include arabinan, galactomannan, chitin, pectin and hyaluronan (Fig. 4).

**Table 5:** List of surface layer homology domain containing proteins of Pjdr2 proposed to be involved in extracellular polysaccharide processing

LT^a^	Domain architecture^b,c,d^	Secretion^f^	MW (kDa)^g^	pI^g^	Putative function^h^
**0221/ Xyn10A**_**1**_	3CBM4,9/GH10/CBM9/3SLH	Yes	157	4.90	β-xylanase
0680	GH5/FN3/3CBM11/3SLH	Yes	204	4.72	β-mannanase
0824	3SLH/GH16/3CBM4,9/DUF1533/GH16/3CBM4,9/DUF1533/CBM4,9/DUF1533/CF/DUF1533/CF	Yes	313	4.65	β-glucanase
**0951/ Bgl16A**_**1**_	3SLH/GH16/2CBM4,9/CBM6/2CBM4,9	Yes	154	4.83	β-glucanase
0964	RBT/2CBM6/RHB/3SLH	Yes	228	4.81	mycodextranase
1124	GH43/CBM6/2BIG2/2SLH	Yes	159	4.71	α-L-arabinofuranosidase
1125	GH20/CBM6/BIG2/2SLH^e^	Yes	213	4.75	α-glucuronidase
1167	DUF481/RHB/FN3/CBM9/FN3/3SLH	Yes	185	4.79	polysaccharide lyase
1173	2PL3/CBM9/3SLH	Yes	210	4.81	pectate lyase
1611	CCT/PHP/BIG3/3SLH	Yes	230	4.90	chitobiase (CCT/ESD) association
1796	2FN3/GH18/FN3/3SLH	Yes	128	4.79	chitinase
1997	CBM4,9/AL3/HP/3SLH	Yes	229	4.90	alginate lyase/heparinase
2544	2NVS/2FN3/3SLH	Yes	170	4.99	sialidase
3195	GAG/3SLH	Yes	153	4.85	hyaluronate lyase
3554	GH30/CBM6/CBM4,9/2FN3/BIG2/3SLH	Yes	173	4.89	endo-1,6-beta-glucosidase
3565	3KCH/2CCT/5BIG3/CLD/3SLH	Yes	227	4.65	CCT/ESD association
4054	2CBM6/PL3/RHB/3SLH	Yes	237	4.75	polysaccharide lyase
4093	3FN3/GH43/CBM6/3SLH	Yes	200	4.70	β-xylosidase
4104	GH27/3CBMX2/3SLH	Yes	164	4.73	α-galactosidase
4665	GH59/CBM6/BIG4/BIG3/3SLH	Yes	217	4.52	β-galactosidase
4730	GH53/BIG4/2CBM4,9/3SLH	Yes	122	4.73	arabinogalactan endo-β-1,4-galactanase
5040	GH66/5CBM6/AMY/3SLH	Yes	182	4.83	dextranase
5076	RHB/2CF/FN3/GH65/2CF/3SLH	Yes	273	4.77	alpha-L-fucosidase
**5200/ Amy13A**_**1**_	4ESD /AMY/2FN3/3SLH	Yes	235	4.75	amylopullulanase
5272	2CBM11/BIG2/3SLH	Yes	231	4.65	carbohydrate binding
5379	3SLH/GH18	Yes	59	5.80	peptidoglycan hydrolase
5534	RHB/2CRD/2CLD/BIG2/3SLH	Yes	176	4.58	polysaccharide lyase
5572	GH42/3SLH	Yes	162	4.72	β-galactosidase
6195	3PBX/3SLH	Yes	77	4.53	xylanase

^a^LT, locus tag annotated as Pjdr2_#### abbreviated to consist only of the numeric portion, ####. Surface anchored proteins directly involved in the utilization of xylan, barley β-glucan and starch are denoted in bold

^b^Domain predictions result from analysis of the proteins in the CCD (Conserved Domain Database) with an Expect Value threshold set to the default of 0.010 and increased to 0.10 to detect more divergent domains in unaccounted for regions or, as in some cases directly through the pfam database. Domain abbreviations are defined in order of appearance. *CBM*, Carbohydrate binding module; *GH*, Glycoside hydrolase; *SLH*, Surface layer homology domain; *FN3*, Fibronectin type 3 domain; *DUF*, Domain of unknown function; *CF*, Coagulation factor 5/8 C-terminal domain; *RBT*, Ricin-type beta-trefoil; *RHB*, Right handed beta helix; *BIG*, Bacterial Ig-like domain; *PL*, Pectate lyase; *CCT*, Chitobiase/beta-hexosaminidase C-terminal domain in the early set domain superfamily; *PHP*, Polymerase and histidinol phosphatase domain; *AL*, Alginate lyase; *HP*, Heparinase; *NVS*, Non-viral sialidases; *GAG*, Glycosaminoglycan polysaccharide lyase family; *KCH*, Galactose oxidase central domain; *CLD*, Cadherin-like beta sandwich domain; *AMY*, Alpha amylase catalytic domain family; *ESD*, Early set domain; *CPRD*, Carboxypeptidase regulatory-like domain; *PBX* Putative bacterial xylanases

^c^For any given domain an abbreviation is provided as defined under superscript (b), with numbers preceding the abbreviation indicating the number of consecutive occurrences greater than one (> 1) of the domain and numbers following the abbreviation indicating the specific family of the detected domain, if any

^d^In each modular protein the domain used to establish the “putative function” (sixth column) is underlined

^e^Annotated in the CAZy database as a GH115

^f^Secretion was deduced by detection of a signal peptide using the Signal-P server

^g^Molecular weight (MW) and isoelectric point (pI) predictions were obtained through the ProtParam tool available through the ExPASy web server

^h^Putative function is based either on the predicted function from domain assignment or a justification for assignment as a protein that is involved with sugar manipulations

### Polysaccharide utilization in Pjdr2

From the studies presented here for the utilization of barley-derived β-glucan and starch, we observe a similar strategy evolved by Pjdr2 as illustrated in the earlier xylan transcriptome report [1]. In each case coordinately expressed gene sets have been identified (Fig. 1) and central to each encoded enzyme system is a multimodular glycoside hydrolase containing carbohydrate binding modules which afford interaction with polysaccharide substrates and a triplicate set of SLH domains for cell surface localized formation of oligosaccharides (Fig. 4).

For processing of β-1,3(4)-glucans, contiguous genes encoding transcriptional regulators, ABC transporters, the multimodular cell-associated Bgl16A_1_ and the secreted non-modular Bgl16A_2_ catalytic domain along with an associated ABC transporter comprise a β-glucan utilization regulon. In this case both secreted enzymes digest barley β-glucan to tri-, tetra-, penta- and hexasaccharides, and laminarin to mono-, di-, tri- and tetrasaccharides indicating similar functions for both enzymes [18]. These oligosaccharides resulting from extracellular barley β-glucan hydrolysis and cellobiose (from either barley β-glucan or growth on cellobiose) are presumably transported into the cell where they are subsequently degraded to monosaccharides by the action of a GH3 endoglucanase and/or a novel GH43 enzyme (Xyn43B_2_) with β-glucosidase functionality.

For starch processing, a regulon encoding a putative maltodextrin ABC transporter together with the non-modular Amy13A_1_ managed starch utilization. Encoded distally, the multimodular cell-associated amylase Amy13A_2_ likely produces small maltodextrins proximal to the cell surface. These may then be taken up and processed by intracellular maltogenic Amy13A_3_ to yield maltose and glucose. Final conversion of maltose is thought to occur through the action of an α-glucan phosphorylase yielding glucose and glucose-1 phosphate.

For the soluble β-glucan, starch and xylan utilization systems, two endo-acting hydrolases may work synergistically with each other for efficient depolymerization of the specific polymeric substrate to oligosaccharides. The modular property of the larger enzyme allows generation of oligosaccharides close to the cell surface without diffusion into the medium and hence couples the depolymerization process with assimilation by ABC transporters for intracellular processing and metabolism. These systems for polysaccharide utilization with minimized secretion of extracellular glycoside hydrolases coupled to transport of oligosaccharides in lieu of simple monomeric sugars potentially affords a significant conservation of cellular energy in the form of ATP as described for the processing of cellulose by *C. thermocellum*.

Based on increased expression levels of genes during growth on multiple polysaccharides a regulatory connection is observed between utilization of barley β-glucan, starch and xylans. Barley β-glucan induces genes involved in extracellular depolymerization and assimilation specific to soluble β-1,3(4)-glucan. However, it also induces many of the genes shown to play a prominent role in the xylan utilization systems [1]. Although xylans do not induce genes specific to barley β-glucan utilization, they do induce genes belonging to the starch utilization system. When Pjdr2 was grown on starch, no genes specific to xylan or barley β-glucan utilization were found to be induced. These studies show the transcriptional induction and repression strategies evolved in Pjdr2 for utilizing a variety of polysaccharides.

Interestingly, induction of the starch utilization genes with growth on xylan results in increased expression of *amy13A*_*1*_ and *amy13A*_*3*_ while the *amy13A*_*2*_ gene encoding the large surface anchored amylase is expressed just enough to meet the significance selection cutoff (4-fold). This same pattern is also observed with growth on maltose. It appears the elevated expression of the *amy13A*_*2*_ gene is specific for starch and the non-starch substrates which activate the expression of the starch utilization regulon (including *amy13A*_*3*_) may poise Pjdr2 for rapid response to starch availability.

SLH domains appear to play a vital role in interaction of Pjdr2 with its native environment. The 77 SLH domain-containing proteins encoded in the genome of Pjdr2 highlight the expanded use of this domain for cell wall associations and also hints to a *modus operandi*, at least regarding an approach to polymeric substrate utilization.

## Conclusion

The genome of Pjdr2 comprises genes encoding extracellular cell-associated depolymerizing enzymes to bioprocess various plant polysaccharides and these include xylans, soluble β-glucans, starch, and also arabinans and galactomannans (Fig. 4). The polysaccharide utilization systems in Pjdr2 serve as potential candidates for further evaluation or for introduction into other related fermentative bacteria to serve as biocatalysts to achieve direct conversion of non-cellulosic biomass to desired products. Preliminary studies have shown the ability of Pjdr2 to produce fermentative products including lactate, acetate, and ethanol from xylans, β-1,3(4)-glucans, and starch, under oxygen limiting conditions (unpublished). The potential of Pjdr2 to produce individual cell-associated glycoside hydrolases for processing non-cellulosic polysaccharides is an alternative strategy to the cell-associated cellulosome complexes evolved by cellulolytic *Clostridium* [14]. Pjdr2 may be considered for direct bioprocessing of hemicelluloses or may be co-cultured with cellulolytic organisms tolerant of microaerophilic conditions for conversion of biomass to targeted products. Pjdr2 is a candidate for further development as a biocatalyst for consolidated bioprocessing of biomass derived from energy crops and agricultural residues to targeted biofuels and chemicals.

## Methods

### Reagents

The carbohydrates xylose, arabinose, glucose, maltose and cellobiose were of the highest purity available. The starch was purchased from Sigma-Aldrich (St. Louis, MO, USA) and was reported to be pure. Soluble low-viscosity barley β-glucan (Product No. P-BGBL, Lot 100402a) was purchased from Megazyme International (Wicklow, Ireland) and was reported to contain < 0.1 % arabinoxylan and < 0.31 % starch. The SG and SO xylans were purified from ground sweetgum wood [2, 43] and sorghum stalk bagasse [7] as previously described [44, 45] using standard procedures.

### Growth of Pjdr2

Pjdr2 was routinely cultivated and growth for RNA isolation as described previously [1–4]. A total of 11 growth conditions were considered in this study. These include barley β-glucan (B) and starch (S) along with their representative dimeric and simple sugars cellobiose (C), maltose (M) and glucose (G), respectively. The sample preparation and RNA-seq data acquisition portion of this manuscript overlaps with a recently published xylan utilization transcriptome [1] which studied the polysaccharide and simple sugar substrates sweetgum wood glucuronoxylan (SG), sorghum stalk glucuronoarabinoxylan (SO), xylose (X) and arabinose (A). Together these paired studies included yeast extract (YE) and sweetgum wood glucuronoxylan without YE supplementation (SGnoYE) as control growth conditions for a total of eleven conditions studied. The characteristics of all growth conditions were defined prior to RNA studies to determine the early mid-exponential phase for harvesting the cells for RNA isolation. Cells were inoculated into 2 ml of 1 % yeast extract (YE) with Zucker-Hankin (ZH) [46] salt medium in 16×100 mm culture tubes and grown overnight at 30 °C, with an orbital rotation of 250 rpm or with a Roto-torque positioned at a 45° angle set at high mode and speed 8. After 24 h the optical density at 600 nm (OD_600_) was measured and cells were harvested (13,000 rpm, 1 min) to start a sub-culture with 2 % inoculum in 15 ml of 1 % YE in ZH medium in a 250 ml flask. These cultures were grown at 30 °C and 250 rpm using a G-2 gyrotary shaker (New Brunswick Scientific) for approximately 6 h to an OD_600_ of 0.5–0.8. The cells were harvested to make an initial inoculation with starting OD_600_ of 0.04 in 15 ml of desired growth media for this study. These sample cultures were then grown at 30 °C and 250 rpm until the empirically predetermined early to mid-exponential harvest time for a given condition, generally OD_600_ 0.4–0.8 for carbon supplemented and 0.25 for yeast extract. Growth conditions for RNA sequencing (RNA-seq) transcriptomic analysis consisted of ZH media with 0.5 % carbohydrate and 0.5 % YE, except in two control conditions. The YE control consisted of 0.5 % YE with no carbohydrate and SG control (SGnoYE) with 0.5 % sweetgum GX_n_ in ZH medium without YE. The culture aliquots used for RNA isolation were streaked onto xylan agar plates to confirm the purity of the cultures.

### RNA isolation

For each of the 11 growth conditions, three parallel cultures were grown for a total of 33 RNA isolations. The amount of cells harvested from each culture was determined through empirical analysis of previous growth studies. Total RNA was isolated from early mid-exponential growing cultures using the RNeasy Protect Bacteria Mini Kit from Qiagen (Valencia, CA, USA) without the use of the RNA Protect reagent. Cells were lysed according to protocol four in the RNAprotect Bacterial Reagent Handbook (2nd edition) with application of lysozyme and Proteinase K as instructed. RNA was purified from the resulting cell lysate using the RNeasy column (protocol seven of the handbook) with on-column RNase-free DNase treatment to remove traces of DNA (Appendix B of the handbook) or in some cases using the TURBO-DNA-*free* kit from Ambion (Life Technologies, Carlsbad, CA, USA). Total RNA was quantified by absorbance at 260 nm and the purity was assessed with the 260/280 nm absorbance ratio. Absence of DNA in the RNA preparations was verified by PCR. In some cases the RNeasy on-column DNase treatment was applied and treated again if required.

The RNA preparations were submitted for Bioanalyzer (Agilent, Santa Clara, CA, USA) analysis at the University of Wisconsin Biotechnology Center or The University of Florida Interdisciplinary Center for Biotechnology Research to verify the absence of RNA degradation. The RNA specific quantification of the samples was performed with the Qubit fluorimeter (Life Technologies, Carlsbad, CA) prior to sample submission to the Joint Genome Institute (JGI), Walnut Creek, CA,.

### RNA sequencing and data analysis

RNA sequencing was performed by the Joint Genome Institute (JGI), US Department of Energy, Walnut Creek, CA, as previously described [1]. Briefly, rRNA-depleted RNA was fragmented using divalent cations and high temperature. Fragmented RNA was reverse transcribed using random hexamers and Superscript II (Invitrogen) followed by second strand synthesis. The fragmented cDNA was treated to allow end-pair A-tailing adapter ligation and 10 cycles of PCR. Libraries were quantified by qPCR. The libraries were sequenced using the Illumina HiSeq sequencing platform utilizing a TruSeq paired-end cluster kit, v3, and 161 Illumina’s cBot instrument to generate clustered flow cells for sequencing. Sequencing of the flow cells was performed on the Illumina HiSeq2000 sequencer using a TruSeq SBS sequencing kit 200 cycles, v3, following a 2×100 indexed run recipe. Raw sequence read data was filtered using BBDuk (filterk = 27, trimk = 27; https://sourceforge.net/projects/bbmap/) to remove Illumina adapters, known Illumina artifacts, phiX, trim Illumina adapters from the right end of the read and quality-trim the right end of the read to Q6. Resulting reads containing one or more ‘N’, or with quality scores (before trimming) averaging less than 10 over the read, or length under 33 bp after trimming, were discarded.

The filtered raw data files were analyzed using ArrayStar ver. 12.2 software from DNASTAR (Madison, Wisconsin). The results from all growth conditions (in triplicates) were mapped to the annotated genome, averaged and normalized across the entire 11 conditions for comparisons. ANOVA analysis was performed to assess gene data quality with respect to the global dataset. The final output was provided as RPKM values (*R*eads *P*er *K*ilobase per *M*illion reads sequenced). In ArrayStar, statistical analysis for comparisons of two conditions was performed with the moderated *t*-test and adjusted *p*-values were calculated using the FDR (Benjamini Hochberg) method [47]. Unless otherwise stated, the expression of genes discussed in this study is based upon a fold difference relative to YE control. Data with *p*-values less than 0.05 were considered to be significant.

### Gene annotation and analysis

Functional roles were assigned to genes based on analysis by BLASTp from NCBI (http://www.ncbi.nlm.nih.gov/) [48, 49], IMG database (http://img.jgi.doe.gov/) or Pfam (http://pfam.xfam.org/) [50]. For genome analysis of the SLH containing genes, the first and third SLH domains of xylanase XynA1 were blasted against the genome and the two resulting datasets combined and made nonredundant. Operon predictions were based on *in silico* analysis using the PePPER webserver (http://server.molgenrug.nl/) [51]. The genes from the genome of Pjdr2 are identified by their locus tags. The locus tags are identified as Pjd2_####, where #### represents the 4-digit gene number used for gene identification in this study. The filtered raw data was processed using ArrayStar. This processed data is available in the supplemental material (Additional file 2). The data represented in the Tables have expression data rounded off to 1 or 2 decimal points, and the *p*-values converted to 2 decimal points. Raw data is available as described in Availability of Supporting Data section.

### Availability of supporting data

The data sets supporting the results of this article are available in the JGI Genome Portal repository, Project ID 1023680 located at http://genome.jgi.doe.gov/pages/projectStatus.jsf?db=Paespnscriptome.

## Additional files

Additional file 1:
**A table summarizing the proteins belonging to the xylan utilization systems in Pjdr2 as previously determined [** 1 **].** (DOCX 19 kb) Additional file 2:
**Total transcriptome data representing all growth conditions from this study.** The data table consists of Pjdr2 gene locus tag ID (accession numbers), *in silico* annotation results, per transcript ANOVA analysis results, calculated transcript quantity given as RPKM values (*R*eads *P*er *K*ilobase per *M*illion reads sequenced), fold change data for each gene in every growth condition relative to the YE control with corresponding *p*-values (moderated *t*-test), fold change data for each gene in the glucan containing growth conditions relative to their constituent sugars with corresponding *p*-values and fold change data for each gene in the xylan growth conditions relative to xylose and arabinose with corresponding *p*-values. Analyses were performed using DNASTAR Arraystar v12 (Madison, WI). (XLSX 6349 kb)
