# Supplementary material for: Genomic and transcriptomic analysis of carbohydrate utilization by Paenibacillus sp. JDR-2: systems for bioprocessing plant polysaccharides
Source: BMC Genomics. 2016 Feb 24;17:131. doi: 10.1186/s12864-016-2436-5 (PMC4765114; doi:10.1186/s12864-016-2436-5)
Supplement: Additional file 1: — A table summarizing the proteins belonging to the xylan utilization systems in Pjdr2 as previously determined [ 1 ]. (DOCX 19 kb) [file 12864_2016_2436_MOESM1_ESM.docx]

**Additional File 1:** Proteins belonging to the xylan utilization systems in Pjdr2

| **Family^a^** | **LT^b^** | **Assigned Protein Product^a^** | **Assigned Name^c^** |
| --- | --- | --- | --- |
| Extracellular | | | |
| GH11 | 4664 | endo-1,4-β-xylanase (secreted) | Xyn11 |
| GH10 | 0221 | endo-1,4- β-xylanase (extracellular cell-associated) | **Xyn10A_1_** |
| GH43 | 4267 | arabinoxylan arabinofuranohydrolase (secreted) | Axh43 |
| Intracellular | | | |
| GH115 | 5977 | α-glucuronidase | **Agu115** |
| GH67 | 1323 | α-glucuronidase | **Agu67A** |
| GH10 | 1324 | endo-1,4- β xylanase | **Xyn10A_2_** |
| GH43 | 1325 | xylan 1,4- β-xylosidase | **Xyn43B_1_** |
| GH43 | 0750 | xylan 1,4- β-xylosidase | Xyn43B_2_ |
| GH43 | 1907 | xylan 1,4- β-xylosidase | Xyn43B_3_ |
| GH51 | 3599 | α-N-arabinofuranosidase | **Abf51B** |
| GH10 | 4260 | endo-1,4- β-xylanase | Xyn10A_3_ |
| GH8 | 1182 | exo-oligoxylanase (reducing end) | Xyn8 |
| CE1 | 4290 | esterase | Est1 |
| Transporters | | | |
| ABC | 1320 | extracellular SBP | UgpB |
|  | 1321 | BPD transport system IMP | LplB |
|  | 1322 | BPD transport system IMP | UgpE |
| ABC | 0728 | extracellular SBP | Abc2x |
|  | 0729 | BPD transport system IMP | Abc2y |
|  | 0730 | BPD transport system IMP | Abc2z |
| ABC | 1809 | BPD transport system IMP | Abc3x |
|  | 1810 | BPD transport system IMP | Abc3y |
|  | 1811 | extracellular SBP | Abc3z |
| ABC | 0661 | extracellular SBP | Abc4x |
|  | 0662 | NBD | Abc4y |
|  | 0663 | IMP | Abc4z |
| ABC | 5273 | BPD transport system IMP | Abc5x |
|  | 5274 | BPD transport system IMP | Abc5y |
|  | 5275 | extracellular SBP | Abc5z |
| ABC | 5314 | BPD transport system IMP | Abc6x |
|  | 5315 | BPD transport system IMP | Abc6y |
|  | 5316 | extracellular SBP | Abc6z |
| ABC | 5596 | BPD transport system IMP | Abc7x |
|  | 5597 | BPD transport system IMP | Abc7y |
|  | 5598 | extracellular SBP | Abc7z |

^a^ GH, glycoside hydrolase; CE, carbohydrate esterase; ABC, ATP binding cassette; SBP, solute binding protein; IMP, inner membrane protein; BPD, binding protein dependent; NBD, nucleotide binding domain.

^b^ LT, locus tag annotated as Pjdr2_#### abbreviated to only consist of the numeric portion, ####.

^c^ The protein name assigned to the xylan utilization gene with those characterized in our laboratory in bold.
